# Deep Learning-Based Identification of Maize Leaf Diseases Is Improved by an Attention Mechanism: Self-Attention

**DOI:** 10.3389/fpls.2022.864486

**Published:** 2022-04-28

**Authors:** Xiufeng Qian, Chengqi Zhang, Li Chen, Ke Li

**Affiliations:** ^1^School of Information and Computer, Anhui Agricultural University, Hefei, China; ^2^Anhui Provincial Engineering Laboratory for Beidou Precision Agriculture Information, Anhui Agricultural University, Hefei, China; ^3^Information Materials and Intelligent Sensing Laboratory of Anhui Province, Anhui University, Hefei, China; ^4^School of Plant Protection, Anhui Agricultural University, Hefei, China

**Keywords:** crop disease, machine learning, deep learning, attention mechanism, neural network

## Abstract

Maize leaf diseases significantly reduce maize yield; therefore, monitoring and identifying the diseases during the growing season are crucial. Some of the current studies are based on images with simple backgrounds, and the realistic field settings are full of background noise, making this task challenging. We collected low-cost red, green, and blue (RGB) images from our experimental fields and public dataset, and they contain a total of four categories, namely, southern corn leaf blight (SCLB), gray leaf spot (GLS), southern corn rust (SR), and healthy (H). This article proposes a model different from convolutional neural networks (CNNs) based on transformer and self-attention. It represents visual information of local regions of images by tokens, calculates the correlation (called attention) of information between local regions with an attention mechanism, and finally integrates global information to make the classification. The results show that our model achieves the best performance compared to five mainstream CNNs at a meager computational cost, and the attention mechanism plays an extremely important role. The disease lesions information was effectively emphasized, and the background noise was suppressed. The proposed model is more suitable for fine-grained maize leaf disease identification in a complex background, and we demonstrated this idea from three perspectives, namely, theoretical, experimental, and visualization.

## Introduction

Maize is one of the most important crops for humanity, with the highest yield globally (Ranum et al., 2014). Maize diseases can cause severe yield reductions, a critical problem (Savary et al., 2012). Therefore, it is vital to promptly identify and monitor maize diseases during the growing period. Accurate identification of diseases in maize is difficult for crop growers who may not be professional in plant pathology, and expert identification is expensive and time-consuming (Ouppaphan, 2017). Traditional image recognition methods and deep learning are gradually entering the field of plant disease recognition (Saleem et al., 2019).

Mobile terminals based on web services and support vector machine (SVM) as back-end algorithms can automatically identify maize diseases (Zhang and Yang, 2014). Zhang et al. (2015) proposed an improved genetic algorithm-SVM (GA-SVM) algorithm to improve the accuracy. A recent study on maize disease identification compared five standard machine learning methods (Panigrahi et al., 2020), namely, Naive Bayes (NB), Decision Tree (DT), K-Nearest Neighbor (KNN), SVM, and Random Forest (RF), with RF achieving the highest accuracy of 79.23%.

However, traditional machine learning is mainly limited by feature extraction and feature representation. Deep learning has made significant progress in plant disease identification (Liu and Wang, 2021). Since AlexNet was proposed in 2012 (Krizhevsky et al., 2012), convolutional neural networks (CNNs) have been widely used in academia and industry, e.g., face detection in dangerous situations (Wieczorek et al., 2021) and combination of Internet of things (IoT) and pearl millet disease prediction (Kundu et al., 2021). In the field of plant disease identification, Dhaka et al. (2021) provided a systematic review of relevant deep learning techniques. Due to its low complexity, a lightweight CNN for mobile terminals has achieved satisfactory performance in maize disease identification (Ouppaphan, 2017). A CNN-based system (DeChant et al., 2017) was implemented to automatically identify northern leaf blight, addressing the challenges of limited data and various irregularities appearing in field-grown images. Ahila Priyadharshini et al. (2019) proposed a CNN modified from LeNet for identifying four maize categories (three diseases classes and one health class) with an accuracy of 97.89%.

However, most of the current studies are based on simple background maize leaf or other crop disease recognition, and the recognition effect of the trained models deteriorates in real field settings, because background noise information causes serious obstruction (Lv et al., 2020). Current research on popular or novel deep learning image recognition algorithms (CNNs) is mainly tested on the public dataset ImageNet, and its images are different from fine-grained images of crop disease. Those designed CNNs mostly focus on patterns of objects in images (e.g., profile features of dogs or cats), and these pattern features are reflected in feature maps of convolutional output, as can be demonstrated by numerous neural network visualization studies (Chattopadhay et al., 2018; Chen et al., 2020; Jiang et al., 2021). In contrast, fine-grained crop disease lesions are usually similar and discrete on the leaf surface; thus, CNNs may not be fully adapted to fine-grained maize leaf disease image classification tasks, which will result in no increase in model performance even by stacking the network layers and increasing model parameters. Rational model design for specific tasks is important and necessary, and the following analysis and experiments in this article also prove this perspective. In addition, many visual disturbances (e.g., reflection, dispersion, and blur) seriously affect fine-grained image classification (Lu Y. et al., 2017; Yang et al., 2020). Therefore, fine-grained maize disease identification in complex background field settings requires more rational models and computerized mechanisms.

Mutual attention between words is highly essential for machine translation tasks, which determines whether a sentence can be translated accurately. The transformer architecture (Vaswani et al., 2017) with the attention mechanism has achieved significant success in natural language processing (NLP). Although previous attention mechanisms have been applied to some specific tasks, e.g., image caption generation technology (Lu J. et al., 2017), text classification (Li et al., 2019), and human action recognition (Song et al., 2017), the form and principle of their attention mechanisms are too different and specialized. However, the transformer’s attention mechanism (self-attention) has a universal form.

**FIGURE 1:**
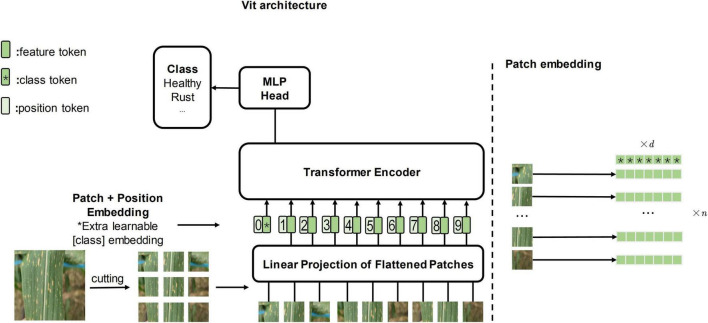
The left side of the figure is the original vision transformer architecture, and the illustration is inspired by Dosovitskiy et al. (2020). The right side of the figure is the patch embedding method which cuts the image into several patches.

To explore whether the attention mechanism will bring enhancements to the field of computer vision, vision transformer (ViT, Figure 1 depicts it) (Dosovitskiy et al., 2020) applies the transformer architecture directly to image classification tasks for the first time, outperforming the state of the art on large-scale datasets. Subsequently, researchers gradually began to study ViT and its attention mechanism. Transformer in transformer (TNT) (Han et al., 2021) embeds the inner transformer into the outer transformer to improve the feature extraction capability lacking in the patch embedding method (refer to Figure 1 for the patch embedding method). Compact convolutional transformer (CCT) (Hassani et al., 2021) demonstrates that convolution can be used to extract local information better, thus making it possible to apply transformer to more tasks with small datasets.

**FIGURE 2:**
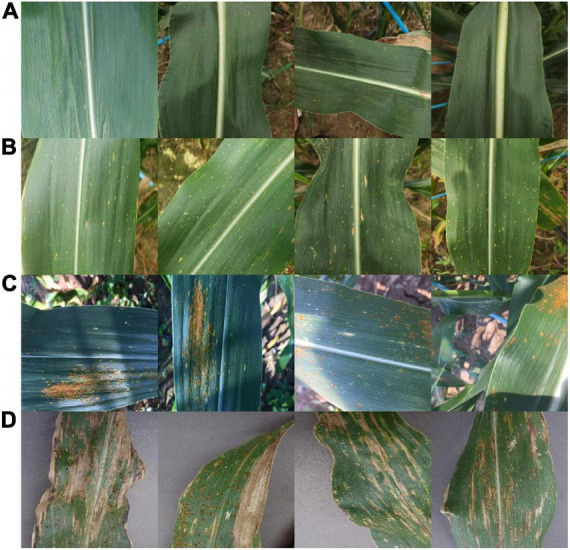
Showing four categories of samples of maize. **(A)** H. **(B)** SCLB. **(C)** SR. **(D)** GLS.

In this study, we found that transformer and self-attention computer mechanisms are more suitable for maize leaf disease identification in complex backgrounds. This article will demonstrate their efficiency and why they work from three perspectives, namely, theoretical derivation, experiment, and visualization. We collected maize leaf diseases datasets with complex backgrounds in our experimental field and proposed an improved model based on ViT and CCT to classify maize into four categories (Figure 2), namely, healthy (H), southern corn leaf blight (SCLB) (Aregbesola et al., 2020), gray leaf spot (GLS) (Saito et al., 2018), and southern corn rust (SR) (Wang S. et al., 2019). The model outperforms some mainstream CNNs compared with it in all metrics, with a smaller number of parameters. In addition, we also conducted experiments on the necessity of the self-attention for the model, demonstrating that it is essential. This article also conducts experiments to observe the effect of the ratio of train set to validation set on the accuracy of the model.

The rest of this article is organized as follows. The section “Materials and Methods” introduces the details of our experimental field and experimental sample cultivation, describing our datasets and methods used to collect them. In that section, we focused on describing our algorithm and the detailed theoretical derivation and proving its effectiveness, as well as the experimental visualization schemes (three schemes). All experimental results are described in section “Results.” We discussed the reasons for the efficiency of this model and some possible future extensions in section “Discussion.”

## Materials and Methods

### Data Collection and Preparation

The dataset of the images, which included 7,701 images, consists of two parts, namely, one part is collected from the public dataset Plant Village and the other part is taken by mobile phones in the natural environment of our experimental field. The maize plants grown in the experimental field are used to select suitable disease-resistant varieties, so there are numerous maize varieties. However, as with other studies on maize disease identification, the variety of maize is not the focus and has no impact on the study of this article because the images of maize leaves in our dataset do not reflect their genetic variety. An area of the experimental field covered 3 acres was chosen for this study, planting a total of 80 rows of maize with 26 maize plants per row, 65 cm between rows, and 13 m length of each row. Half of this area was planted with maize inoculated with SCLB, and the other half with maize inoculated with SR. The conidia with a concentration of 10^6^/ml were sprayed at this maize in the sixth-leaf stage, namely, 40–50 days after sowing, to inoculate maize with the abovementioned diseases. After inoculation, the maize is allowed to develop naturally. One day of the milk stage of the maize is chosen to take all the images needed for our dataset. Every maize plant is sampling points. We walked along the rows and remained for several minutes to take images, and the same leaf will be photographed more than once to get 1–6 images. Furthermore, the leaves were manually moved to find a better angle to photograph a good image while adjusting the position of the phones to aid this operation. Despite the fact that a leaf may be photographed more than once, every image is different and contains complicated background visual information because the content of interest is different for each shot. The manual focus is chosen to solve the issue that phones cannot focus on the leaf lesion areas of interest, therefore, guaranteeing every image is clear and focused. The H maize images were obtained from another area of the experimental field where eight rows of maize plants were planted, and the planting pattern and the photographing mode are identical to the above. All the images photographed are under normal uncontrolled lighting conditions with mobile phones’ low-cost red, green, and blue (RGB) sensor. The GLS maize images are downloaded from the Plant Village. This article divided the dataset into a training dataset and a validation dataset according to the principles of 3 to 1 due to the sample balance. Table 1 shows the distribution of images and the division of the dataset.

**TABLE 1:** Distribution of data sources and division of training set and validation set.

Categories	Shooting by us	Plant village	Train set	Validation set
SCLB	2,243	0	1,743	500
H	1,273	1,162	1,953	500
SR	2,023	0	1,523	500
GLS	0	1,000	750	250

### Data Processing

The images’ size must be unified to a standard 224 × 224-pixel square offline to reduce the computational effort before the model training. Furthermore, some data augmentation techniques are separately applied to each image, with a certain probability during model training online, thus enhancing the generalization ability of the model and preventing its overfitting. This article selects four data augmentation techniques suitable for maize leaf disease identification, namely, RandomFlip, ColorJitter, Cutmix (Yun et al., 2019), and Mixup (Zhang et al., 2018). Before an image is imported into the model, RandomFlip randomly rotates it horizontally or vertically, expanding the dataset, as this is equivalent to the images in the dataset having different shooting angles than their raw form. ColorJitter randomly changes the image’s brightness, contrast, saturation, and hue. As a result, ColorJitter can improve the model’s ability to adapt to different lights in field settings. The lesions of the three diseases chosen for the study are scattered on the surface of the leaves, which means that the model should not focus only on the lesions of one area but also on the entire leaf. Cutmix randomly crops a patch of the image and fills the area with a small and same size patch from another image. The size of the patches is a hyperparameter, and the position of the patches on the images is random. Mixup is widely used in image classification tasks, and it mainly constructs a virtual sample $\left(\tilde{\text{x}},\tilde{\text{y}}\right)$ by the following methods:


(1) $$\tilde{\text{x}}=\lambda {\text{x}}_{\text{i}}+\left(1-\lambda \right){\text{x}}_{\text{j}}$$

(2) $$\tilde{\text{y}}=\lambda {\text{y}}_{\text{i}}+\left(1-\lambda \right){\text{y}}_{\text{j}}$$

where x_i_ and x_j_ are two different images, y_i_ and y_j_ are the unique one-hot labels corresponding to these two images, and λ ∈ (0, 1). Mixup extends the distribution of samples by linear interpolation, making it popular for various image classification tasks. Both Cutmix and Mixup make models confusing, forcing them to focus on global information rather than local information. This article’s data augmentation techniques are used only in training, not testing.

### Algorithm

At the beginning of this section, we have done some specifications of mathematical notation and some pre-paving for our model. The upper case non-bolded symbols in this article refer to matrices, the lower case bolded symbols refer to row vectors, and the lower case non-bolded symbols refer to constants or scalar variables. A complete image can be divided into several local regions. The critical feature information of maize leaf disease is located in some local regions where the lesions are located. From the visual point of view, the texture and color of these local areas are the feature information. From the algorithmic point of view, the RGB values of the pixels in these local areas are the feature information. Background information that interferes with the classification is useless information. CNNs usually downsample the image and use the generated feature maps to represent the information of the image. Our model encodes the feature information of local regions into vectors (called tokens) to represent the information of the whole image. The attention mechanism of this article will be based on these tokens to identify those critical regions to make the classification.

**FIGURE 3:**
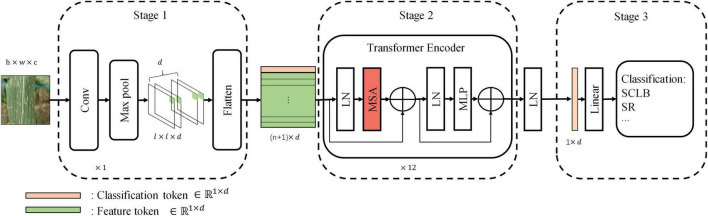
The standard model architecture. Stage 1 extracts information from local regions of the image and encodes them into tokens. Stage 2 is the core computational network that performs the self-attention. Stage 3 maps the classification token into four classes to complete the final classification. Linear, linear layer; LN, layer normalization; MLP, multilayer perceptron.

Our standard model has three stages, namely, Stage 1, Stage 2, and Stage 3 (Figure 3). Stage 1 extracts the image features and encodes them into a feature tokens matrix. Each row vector in the tokens matrix is a token, and a token is a vector used to represent the local visual features within a receptive field (convolution or max-pooling kernel). Passing the input image I ∈ R^h×w×c^ through a convolutional layer and a max-pooling layer generates feature maps *Fm* ∈ ℝ^*l*×*l*×*d*^ with channels of d. The width of feature maps output from both convolution layer and max-pooling layer is expressed by the following equation:


(3) $$l=\frac{i+2p-k}{s}+1$$

where *i* denotes the width of the original image or input feature maps, *k* is the size of the kernel (convolution or max-pooling), *s* is the stride of kernel movement, and *p* is padding. We listed those hyperparameters at the end of the section algorithm. At the end of Stage 1, after extracting vectors along the channel dimension for the feature maps *Fm*, the vectors are arranged to obtain the feature tokens matrix, which can be described by the following equation:


(4) X=Flatten(Fm)

where *X* ∈ ℝ^*n*×*d*^ is the tokens matrix, and *n* = *l*^2^. Each row vector of dimension d in *X* is a feature token.

At the beginning of Stage 2, a learnable vector “classification token” of dimension d is appended to the top of *X*; hence, *X* ∈ ℝ^*n*×*d*^ was transformed to *X* ∈ ℝ^*n*_*t*_×*d*^, where *n*_*t*_ = *n* + 1. The “classification token” is derived from NLP and is similar to BERT’s (Devlin et al., 2018) “class token.” The classification token will be output at the end of Stage 2 as input to Stage 3 to complete the final classification. Therefore, the transformer encoder of Stage 2 is the core computational module of the whole network, and the essential part of it is multi-head self-attention (MSA) that is used to perform self-attention. The rest of the section algorithm will introduce how it works, demonstrating why it is effective. To better explain MSA, we first described the computational process of single-head self-attention (SSA). Tokens matrix *X* is linearly transformed into queries *Q*, keys K, and values V by three matrices, *W_Q_*, *W_K_*, and *W_V_*, respectively, and the linear transforms can be seen in the following equations:


(5) $$Q=XW_{Q}$$

(6) $$K=XW_{K}$$

(7) $$V=XW_{V}$$

where *W*_*Q*_ ∈ ℝ^*d*×*d*^, *W*_*K*_ ∈ ℝ^*d*×*d*^, and *W*_*V*_ ∈ ℝ^*d*×*d*^ are parametric learnable matrices. In fact, each row vector in *Q*, K, and V is still a token used to represent feature information of the corresponding local region. Assume that **q_i_**, **k_i_**, and **v_i_** denote the *i*-th token of *Q*, K, and V, respectively; thus, they all represent the feature information of the *i*-th receptive field of the original image. The correlation between tokens is obtained by calculating the inner product of all row vectors in *Q* and all row vectors in K. For example, $\left\langle {\text{q}}_{i},{\text{k}}_{j}\right\rangle ={\text{q}}_{i}{\text{k}}_{j}^{T}$ represents the correlation between the *i*-th token and the *j*-th token or the degree of attention of the *i*-th token to the *j*-th token. However, it is usually not equal to $\left\langle {\text{q}}_{j},{\text{k}}_{i}\right\rangle ={\text{q}}_{j}{\text{k}}_{i}^{T}$, which is due to two factors. On the one hand, *Q* and K are obtained by a linear transformation of two different learnable matrices, *W_Q_* and *W_K_*. Although both **q_i_** and **k_i_** represent the visual information of the *i*-th receptive field, the elements in *W_Q_* and *W_K_* change in the direction favorable to the final classification as the model weights are updated. On the other hand, the self-attention mechanism is derived from NLP, where words are encoded as vectors (tokens) in a machine translation task. The correct translation of a sentence requires finding the relevance of each word, and two words have different attention to each other, which requires the correlation calculation method between tokens as described earlier. Therefore, the correlation between tokens can be calculated by the following equations:


(8) $$\begin{array}{c} A=QK^{T}\\ =\left[\begin{array}{cccc} {\text{q}}_{1}{\text{k}}_{1}^{T} & {\text{q}}_{1}{\text{k}}_{2}^{T} & \cdots & {\text{q}}_{1}{\text{k}}_{n_{t}}^{T}\\ {\text{q}}_{2}{\text{k}}_{1}^{T} & {\text{q}}_{2}{\text{k}}_{2}^{T} & \cdots & {\text{q}}_{2}{\text{k}}_{n_{t}}^{T}\\ \vdots & \vdots & & \vdots \\ {\text{q}}_{n_{t}}{\text{k}}_{1}^{T} & {\text{q}}_{n_{t}}{\text{k}}_{2}^{T} & \cdots & {\text{q}}_{n_{t}}{\text{k}}_{n_{t}}^{T} \end{array}\right]\\ =\left[\begin{array}{cccc} a_{11} & a_{12} & \cdots & a_{1n_{t}}\\ a_{21} & a_{22} & \cdots & a_{2n_{t}}\\ \vdots & \vdots & & \vdots \\ a_{n_{t}1} & a_{n_{t}2} & \cdots & a_{n_{t}n_{t}} \end{array}\right] \end{array}$$

*A* is the preliminary tokens correlation matrix; in other words, it represents the attention between tokens, e.g., *a*_*ij*_ denotes the attention of the *i*-th token to the *j*-th token or the attention of the visual information of the *i*-th receptive field to the visual information of the *j*-th receptive field. The following equations normalize the attention matrix *A*:


(9) $$\begin{array}{c} A^{{}^{\prime }}=soft\text{max}\left(\frac{A}{\sqrt{d_{k}}}\right)\\ =\left[\begin{array}{c} \sigma \left({\text{a}}_{1}\right)/\sqrt{d_{k}}\\ \sigma \left({\text{a}}_{2}\right)/\sqrt{d_{k}}\\ \vdots \\ \sigma \left({\text{a}}_{n_{t}}\right)/\sqrt{d_{k}} \end{array}\right] \end{array}$$

(10) $$\sigma \left({\text{a}}_{i}\right)=\left[\begin{array}{cccc} \frac{e^{a_{i1}}}{\sum_{j=1}^{n_{t}}e^{a_{ij}}} & \frac{e^{a_{i2}}}{\sum_{j=1}^{n_{t}}e^{a_{ij}}} & \cdots & \frac{e^{a_{in_{t}}}}{\sum_{j=1}^{n_{t}}e^{a_{ij}}} \end{array}\right]$$

where *d_k_* is a normalization factor and a hyperparameter. Assume that α_*ij*_ is the element in row i and column j of *A*′. Subsequently, elements in attention matrix *A*′ are used as weights to linearly combine the tokens of V, which will integrate the information of the tokens they are focused on for each token. The following equation describes this process:


(11) $$\begin{array}{c} V^{{}^{\prime }}=A^{{}^{\prime }}V\\ =\left[\begin{array}{c} \alpha_{11}{\text{v}}_{1}+\alpha_{12}{\text{v}}_{2}+\cdots +\alpha_{1n_{t}}{\text{v}}_{n_{t}}\\ \alpha_{21}{\text{v}}_{1}+\alpha_{22}{\text{v}}_{2}+\cdots +\alpha_{2n_{t}}{\text{v}}_{n_{t}}\\ \vdots \\ \alpha_{n_{t}1}{\text{v}}_{1}+\alpha_{n_{t}2}{\text{v}}_{2}+\cdots +\alpha_{n_{t}n_{t}}{\text{v}}_{n_{t}} \end{array}\right] \end{array}$$

**FIGURE 4:**
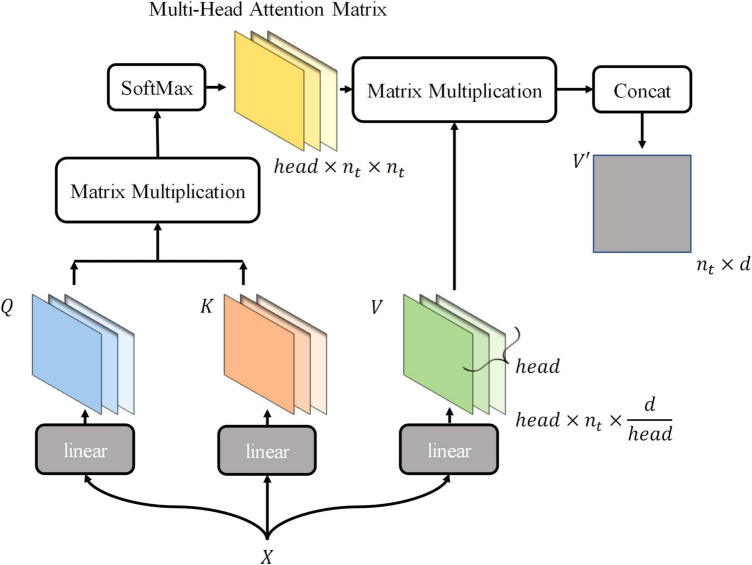
The schematic of implementing multi-head self-attention.

Thus, the new tokens of *V*′ are integrated with the information they pay attention to. The above describes the computation of the attention mechanism. In this process, the classification token is fully involved in the computation of the self-attention mechanism, continuously integrating information about receptive fields in a different-attention way, and finally being output for final classification. The mode using tokens to represent receptive field information and integrating tokens information is more suitable for maize leaf disease identification, because the main characteristic of maize leaf diseases is lesions, which are usually small and widely distributed on the leaf surface. Hence, similarity exists between lesions in terms of texture and color, which is reflected in the RGB values of images. The visual information in receptive fields where lesions exist is similar, and vectors encoded are also similar, so critical information of images can be highlighted by the computational model presented earlier. Subsequent experiments and visualizations in this article will also demonstrate that the model will focus on lesions rather than background noise information. MSA is a simple extension of SSA, performing *head* SSA calculations independently of each other in parallel (Figure 4), and *head* is a hyperparameter. Based on the SSA presented earlier, the MSA is briefly described by the following equations:


(12) $$Q_{i}=XW_{i}^{Q}$$

(13) $$K_{i}=XW_{i}^{K}$$

(14) $$V_{i}=XW_{i}^{V}$$

(15) $$A_{i}^{{}^{\prime }}=soft\text{max}\left(\frac{Q_{i}K_{i}^{T}}{\sqrt{d_{k}}}\right)$$

(16) $$V_{i}^{{}^{\prime }}=A_{i}^{{}^{\prime }}V_{i}$$

(17) $$V^{{}^{\prime }}=Concat\left(V_{1}^{{}^{\prime }},V_{2}^{{}^{\prime }},\cdots ,V_{head}^{{}^{\prime }}\right)=V_{1}^{{}^{\prime }}⊕V_{2}^{{}^{\prime }}⊕\cdots ⊕V_{head}^{\prime }$$

where *i* = 1, 2, ⋯, *head*, $W_{i}^{Q}\in {\mathbb{R}}^{d\times \frac{d}{head}}$, $W_{i}^{K}\in {\mathbb{R}}^{d\times \frac{d}{head}}$, $W_{i}^{V}\in {\mathbb{R}}^{d\times \frac{d}{head}}$, and ⊕ is the concatenated operation to matrices. Therefore, tokens matrix *X* ∈ ℝ^*n*_*t*_×*d*^ is calculated by the MSA and outputs *V*′ ∈ *R*^*n*_*t*_×*d*^.

Layer normalization (LN) (Ba et al., 2016) normalizes input tokens to speed up the convergence by the following equations:


(18) $$LN\left({\text{y}}_{i},\alpha ,\beta \right)=\frac{{\text{y}}_{i}-\mu }{\sigma }⊙\alpha +\beta \in R^{n\times d}$$

(19) $$\mu =\frac{1}{d}\sum_{j=1}^{d}y_{i}^{j}$$

(20) $$\sigma =\sqrt{\frac{1}{d}\sum_{j=1}^{d}{\left(y_{i}^{j}-u\right)}^{2}}$$

where y_i_ is the *i*-th token, and ${\text{y}}_{\text{i}}^{\text{j}}$ refers to the *j*-th element of the *i*-th token. α and β are learnable gains and bias, respectively.

Linear layer can perform a linear transformation of the input matrix, which is described by the following equation:


(21) $$M_{o}=MW+\text{b}$$

where *M* ∈ ℝ^*m*×*n*^ is the input matrix, *W* ∈ ℝ^*n*×*o*^ refers to the learnable matrix, **b** ∈ ℝ^1×*o*^ refers to the learnable bias vector, and *M*_*o*_ ∈ ℝ^*m*×*o*^ refers to the output matrix.

Multilayer perceptron (MLP) obtains nonlinearity and transformation (Han et al., 2021), benefiting from the linear layer and the activation function Gaussian error linear units (GELU) (Hendrycks and Gimpel, 2016). This nonlinear transformation can be described as follows:


(22) $$M_{o}=GELU\left(MW_{1}+{\text{b}}_{1}\right)W_{2}+{\text{b}}_{2}$$

(23) $$GELU\left(x\right)=0.5x\left(1+\text{tanh}\left[\sqrt{2/\pi }\left(x+0.044715x^{3}\right)\right]\right)$$

where *M* ∈ ℝ^*m*×*n*^ and *M* ∈ ℝ^*m*×*o*^ refer to input matrix and output matrix, respectively, *W*_1_ ∈ ℝ^*n*×*h*^ and *W*_2_ ∈ ℝ^*h*×*o*^ are learnable matrices, and **b**_1_ ∈ ℝ^1×*h*^ and **b**_2_ ∈ ℝ^1×*o*^ are learnable bias vectors. The GELU function, when applied to a matrix, will perform a nonlinear transformation on all elements of that matrix.

Stage 3 maps classification token (${\text{v}}_{0}^{{}^{\prime }}$, the first row vector of the matrix *V*′ of the last transformer encoder block) of the output of Stage 2 to four categories by a linear layer.

**FIGURE 5:**
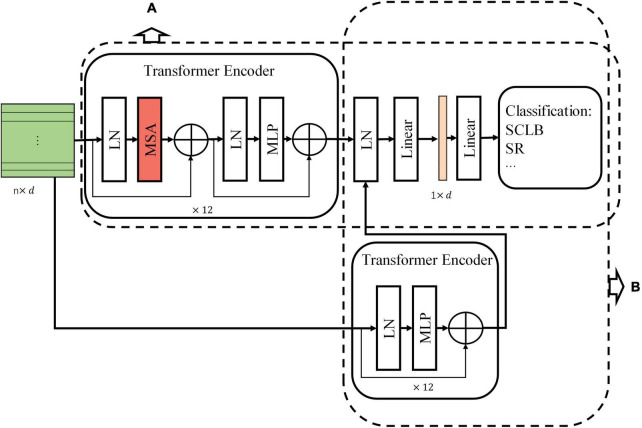
Two models changed from the standard model. **(A)** Model-1. **(B)** Model-2.

We conducted an ablation experiment on MSA to investigate the necessity of self-attention. The experiment needs to remove the MSA from transformer encoder. However, without the MSA, the classification tokens cannot participate in the computation of the integrated tokens information. Therefore, we designed Model-1 and Model-2 based on the standard model. Model-1 does not use classification tokens but integrates feature tokens to classify, and Model-2 removes MSA from Model-1 (refer to Figure 5 for Model-1 and Model-2).

### Hyperparameters and Training Facilities

The hyperparameters of our standard model are as follows:

• Max pool layer: number = 1, kernel size = 3, stride = 2, padding = 1,
• Convolutional layer: number = 1, kernel size = 7, stride = 4, padding = 1,
• Transformer encoder blocks: 12,
• Heads of MSA: 4,
• Dimension of token: d 64,
• Normalization factor: *d_k_* = 16,
• Batch size: 64,
• Learning rate: 0.004,
• Weight decay: 0.05.

We have made our dataset and code, as well as all the trained models of this article, publicly available in the site: https://github.com/haiyang-qian/code-and-dataset. Our model is trained on the open-source deep learning framework Pytorch 1.9, and the programming language is Python 3.7.10. Our experimental facilities are as follows:

• CPU: Xeon Gold 6142
• GPU: RTX 3090
• CUDA: V11.2
• OS: Ubuntu 20.04
• Memory: 60.9 GB
• SSD: 429.5 GB

### Visualization Methods

In this article, three visualization schemes are designed, targeting three network outputs, namely, convolutional or pooling layers, tokens matrix, and classification token for feature tokens’ attention. First, for the feature maps of the output of the convolution or pooling layer, we have applied the Grad-Cam method (Selvaraju et al., 2017). The method first computes gradients for class *c* regarding feature maps *Fm* of a convolutional layer (assume that *Fm^k^* is the k-th channel of the feature maps). These gradients are globally averaged over the corresponding channels of *Fm* to obtain the weights of that channel $\alpha_{k}^{c}$. $\alpha_{k}^{c}$ is the importance of feature map *Fm^k^* for class c and is used to weigh the feature map *Fm^k^*. Then, the class discriminative localization map (CDLM) (a map of the importance of different regions of input image for class c) can be obtained by completing this operation for all the feature maps. The above computation can be described by the following equations:


(24) $$\alpha_{k}^{c}=\frac{1}{z}\sum_{i}\sum_{j}\frac{y^{c}}{Fm_{ij}^{k}}$$

(25) $$L_{Grad-CAM}^{c}=\text{Re}LU\left(\sum_{k}\alpha_{k}^{c}Fm^{k}\right)$$

(26) $$\text{Re}LU\left(x\right)=\left\{ \begin{array}{c} xifx>0\\ 0ifx<0 \end{array}\right.$$

where $L_{\mathrm{Grad}–\mathrm{CAM}}^{c}$ is a CDLM calculated by the Grad-Cam method, and it will be mapped back to the input image to obtain the visualization result. The Grad-Cam method is usually used for feature maps of the convolution or pooling layer output. Therefore, in our second visualization scheme based on the tokens matrix, we have reshaped the two-dimensional feature tokens matrix *Y* into a three-dimensional feature map matrix, expressed as the following mapping:


(27) $$Y\in {\mathbb{R}}^{n\times d}\to Fm\in {\mathbb{R}}^{l\times l\times d}$$

where *n* = *l*^2^. We applied the Grad-Cam method to *Fm* to obtain the results of the second visualization scheme in this article. The third visualization scheme is used to directly map the attention of the classification token to the feature tokens back to the input image. Our standard model has 12 transformer encoder blocks, and each MSA has four heads. The attention of each MSA is combined by the following equations:


(28) $$A^{\left(i\right)}=\sum_{j=1}^{4}A^{ij},i=1,2,\cdots ,12$$

(29) $$A=\sum_{i=1}^{12}\frac{A^{\left(i\right)}-\text{min}\left(A^{\left(i\right)}\right)}{\text{max}\left(A^{\left(i\right)}\right)-\text{min}\left(A^{\left(i\right)}\right)}$$

where *A^ij^* denotes the attention map of classification token to feature tokens in *j*-th head of *i*-th transformer encoder, and *A*^(*i*)^ is the attention map that fuses the attention maps of all the heads in *i*-th transformer encoder. *A* will be mapped directly to the input image. This visualization scheme does not involve any gradient calculation. It will reflect the attention of the classification token to feature tokens and demonstrate whether the calculation of MSA without increasing parameters is effective for identifying diseased maize leaves.

### Evaluation of Model Performance

We chose accuracy, precision, recall, F1 score, parameters, and floating-point operations per second (FLOPs) to evaluate our classification model. Among them, precision, recall, and F1 score can be calculated by the following equations:


(30) $$Precision=\frac{TP}{TP+FP}$$

(31) $$\text{Re}call=\frac{TP}{TP+FN}$$

(32) $$F1=\frac{2Precision\times \text{Re}call}{Precision+\text{Re}call}$$

where *TP* refers to the number of true positives, *FP* refers to the number of false positives, and *FN* refers to the number of false negatives.

## Results

**FIGURE 6:**
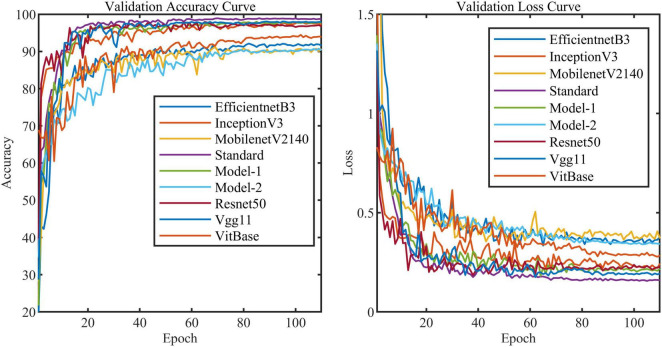
Accuracy curve and loss curve of validation set of all models in this article (i.e., Model-1 and Model-2).

**FIGURE 7:**
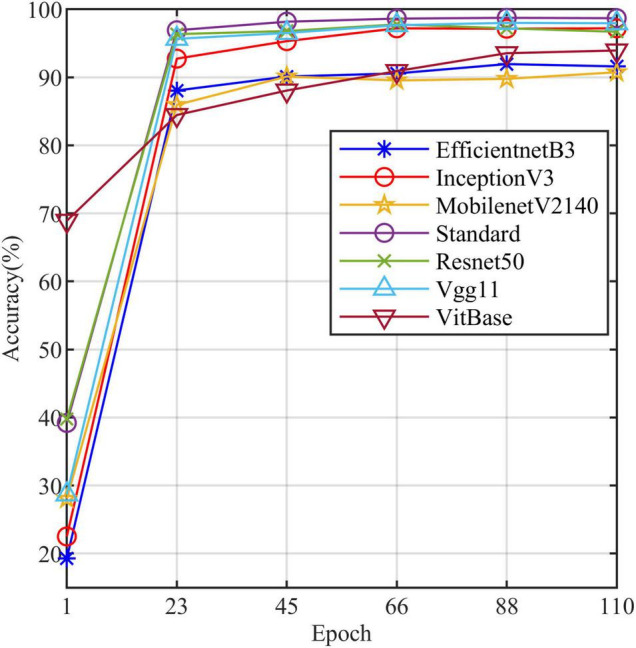
The accuracy line chart of the standard model and the other models.

**FIGURE 8:**
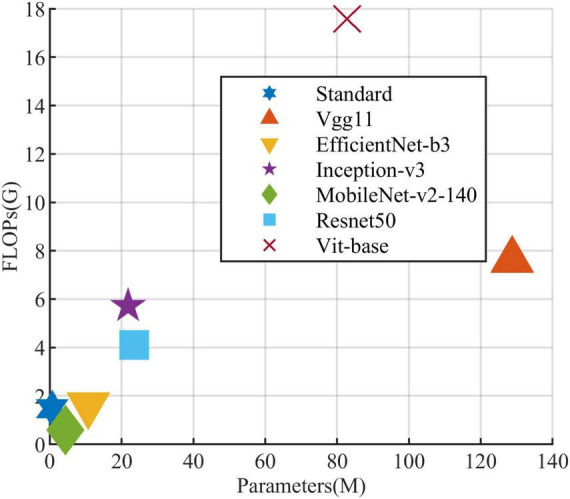
Comparison of the standard model and the other models in parameters and FLOPs.

All models in this article were trained with 110 epochs. Figure 6 shows the accuracy and loss of all models as a function of epochs. As can be seen, the performance dramatically improves within the first 20 epochs, but improvement is minor beyond 20 epochs. We compared five mainstream CNNs with our standard model. These CNNs have achieved excellent performance on some specific tasks. For example, MobileNet (Sandler et al., 2018) can be applied to mobile terminals due to its lightweight architecture. ResNet (He et al., 2016) as a baseline is widely used in the industry. EfficientNet (Tan and Le, 2019) has a relatively significant advantage in terms of speed and accuracy. Table 2 compares the standard model with these CNNs in terms of six metrics (i.e., accuracy, precision, recall, F1 score, parameters, and FLOPs). The accuracies of CNNs reached VGG11 (Simonyan and Zisserman, 2014) 97.9%, ResNet50 96.6%, EfficientNet-b3 91.6%, Inception-v3 (Szegedy et al., 2016) 97.2%, and MobileNet-v2-140 90.7%, whereas the standard model reached 98.7% and surpassed these CNNs. Figure 7 shows that comparison of the accuracy trends of the standard model with the mainstream CNNs and ViT-base during training. The recall of the standard model for class H is 1% lower than that of Vgg11, but it surpasses Vgg11 in all other metrics. Except for VGG11, the standard model surpasses or ties the rest of these CNNs in accuracy, precision, recall, and F1 score. For the FLOPs metric, MobileNet-v2-140 has lower FLOPs than the standard model and requires less computing power. Since MobileNet-v2-140 is designed for mobile terminals, its FLOPs must be lower than common models. Nevertheless, MobileNet-v2-140 has 6.6 times the number of the standard model parameters. The number of parameters and FLOPs of other models are significantly higher than the standard model (Figure 8 clearly shows the comparison of parameters and FLOPs of the models), which means that these CNNs are designed to be bloated for maize leaf disease identification in a complex background. As can be seen, for the specific task of this article, stacking the number of layers of the network and increasing the number of parameters of the model are not effective in improving the performance of the model. Our model has only one convolutional layer and one pooling layer to encode local regions of images into tokens, and transformer encoder as the core computational module, which not only significantly reduces the number of parameters and FLOPs of the model but also achieves the best performance. From another perspective, although the number of parameters of the standard model is on average three orders of magnitude lower than the other models in Table 2, its FLOPs are in the same order of magnitude as theirs. Since MSA involves large-scale matrix computation when computing the attention matrix between tokens, this operation does not involve the model’s parameters but increases the model computation. A comparison in Table 2 between the standard and ViT (accuracy 93.9%) was created to compare the patch embedding method with the convolution method, showing that the convolution method is superior to the patch embedding method from the perspective of results, which indicates that convolutional layer and max-pooling layer can sufficiently encode information of maize leaf disease lesions into tokens and reduce model’s parameters.

**TABLE 2:** Comparison between the standard model and the other mainstream models.

	Standard	VGG11	EfficientNet-b3	Inception-v3	MobileNet-v2-140	ResNet50	Vit-base
Accuracy (%)	98.7	97.9	91.6	97.2	90.2	96.6	93.9
**Precision (%)**							
H	97	96	88	96	88	94	91
SCLB	99	99	90	97	88	99	92
SR	99	98	94	98	92	96	98
GLS	100	100	97	99	99	100	96
**Recall (%)**							
H	99	100	92	98	93	99	95
SCLB	97	96	86	94	86	91	90
SR	100	99	98	100	96	99	97
GLS	99	97	89	98	85	97	92
**F1 (%)**							
H	98	98	90	97	91	97	93
SCLB	98	97	88	95	87	95	91
SR	100	98	96	99	94	98	98
GLS	100	98	93	99	91	98	94
Parameter (M)	0.65	128.78	10.70	21.79	4.32	23.52	82.80
FLOPs (G)	1.47	7.61	1.62	5.72	0.59	4.10	17.58

**FIGURE 9:**
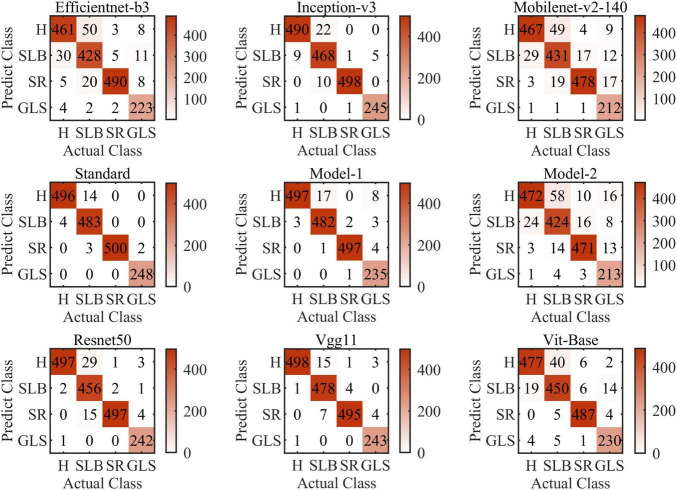
Confusion matrices of all models of this article (i.e., Model-1 and Model-2).

Figure 9 shows the confusion matrices of all models of this article (i.e., Model-1 and Model-2). The confusion matrix’s abscissa axis represents actual class and ordinate axis represents predicted class. As can be seen, for the nine models, they always tend to identify the SCLB class as the H class. SCLB lesions on maize leaves are minor and scattered, which results in some samples infected similar to H class. In contrast, considering computing power limitation, the size of images can be shrunk small, which leads to SLCB lesions-pixels disappearing and classification error. SR and GLS are rarely misclassified, because their symptoms are markedly distinct from other categories of this article. SR lesions on the leaf tissue’s aboveground surface resemble flecks that develop into small golden-brown pustules or bumps. Tan lesions of SR can be distinguished readily from yellow lesions on the surface of maize leaf infected SCLB or GLS.

**FIGURE 10:**
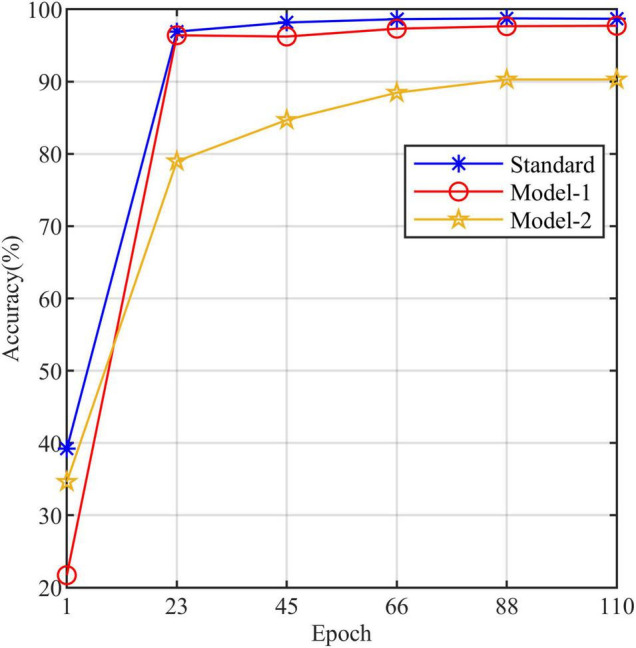
The accuracy line chart of the standard model, Model-1, and Model-2.

Table 3 compares the three models to explore the necessity of the self-attention. Model-1 and Model-2 (Figure 5) are modified from the standard model to conduct this study. Model-1 fuses feature tokens into a classification token in Stage 3 by a linear layer instead of adding a classification token at the end of Stage 1, and Model-2 removes the MSA based on Model-1. Figure 10 clearly shows the increased curve of accuracy of the three models. The accuracy of the standard model exceeds Model-1 by 1%. They have almost the same number of parameters, which indicates that the classification token participating in MSA computation is better than fusing feature tokens into classification tokens. The accuracy of Model-2 is substantially lower than Model-1 by 7.5%. Among other metrics (e.g., precision, recall, and F1 score), Model-2 is also substantially lower than Model-1. The expected results indicate that the self-attention dramatically improves the performance of the model. Model-1 and Model-2 have the same number of parameters, but the FLOPs of Model-2 are much lower than those of Model-1. As mentioned above, the large-scale matrix operations involved in MSA do not increase the number of parameters in the model but do increase the computational complexity of the model. This little computational cost is worth the significant improvement it brings to the model, which also shows that self-attention, a computation that involves almost no parameters of the model, can dramatically improve the identification of maize leaf diseases in complex backgrounds.

**TABLE 3:** Research of importance of the self-attention.

	Standard	Model-1	Model-2
Accuracy (%)	98.7	97.7	90.2
Precision (%)			
H	97	95	85
SCLB	99	98	90
SR	99	99	94
GLS	100	100	96
Recall (%)			
H	99	99	94
SCLB	97	96	85
SR	100	99	94
GLS	99	94	85
F1 (%)			
H	98	97	89
SCLB	98	97	87
SR	100	99	94
GLS	100	97	90
Parameter (M)	0.65	0.66	0.66
FLOPs (G)	1.47	1.46	0.33

**FIGURE 11:**
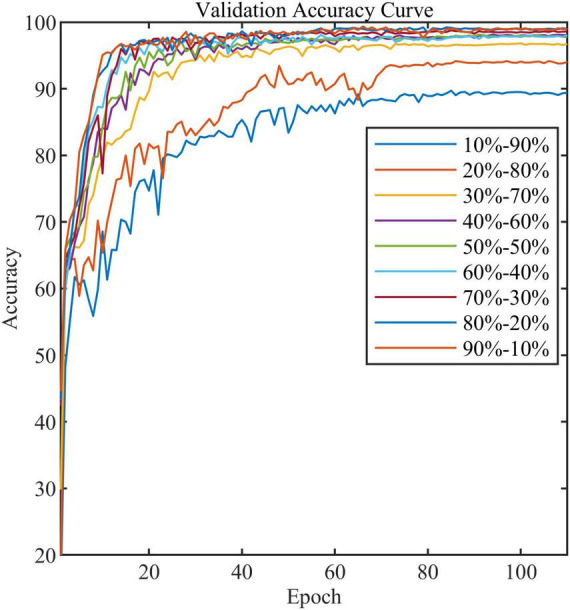
The validation accuracy curve of the standard model in nine train-validation sets.

In addition, we compared the effect of different train and validation set ratios on the accuracy of the standard model (Table 4). As can be seen, the model’s accuracy gradually increases as the ratio increases. When the ratio reaches 20–80%, the accuracy reaches 94.0%, while when the ratio reaches 50–50%, the accuracy almost stops increasing. Figure 11 shows the validation accuracy curve of the standard model over 9 ratios in the training process. The experiment indicates that the standard model can achieve satisfactory performance even when the number of training samples is small.

**TABLE 4:** The standard model accuracy results for each train-validation set.

Train-test split (%)	H	SCLB	SR	GLS	Accuracy
10–90	243/2,192	224/2,019	202/1,821	100/900	0.894
20–80	487/1,948	448/1,795	404/1,619	200/800	0.940
30–70	730/1,705	672/1,571	606/1,417	300/700	0.967
40–60	974/1,461	897/1,346	809/1,214	400/600	0.980
50–50	1,217/1,218	1,121/1,122	1,011/1,012	500/500	0.977
60–40	1,461/974	1,345/898	1,213/810	600/400	0.980
70–30	1,704/731	1,570/673	1,416/607	700/300	0.986
80–20	1,948/487	1,794/449	1,618/405	800/200	0.990
90–10	2,191/244	2,018/225	1,820/203	900/100	0.989
Total	2,435	2,243	2,023	1,000	

**FIGURE 12:**
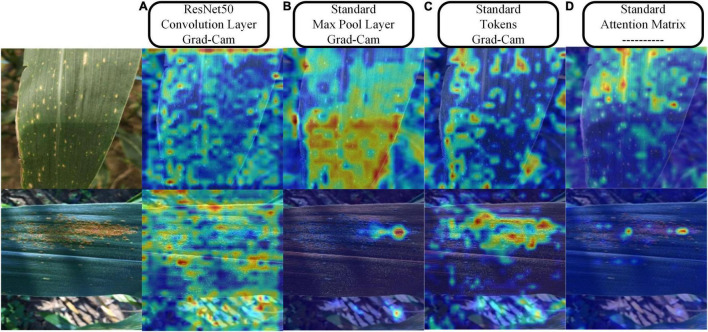
Visualization results of the three schemes. **(A)** Grad-Cam method for visualization of the feature maps output by the Resnet50 convolutional layer. **(B)** Grad-Cam method for visualization of the feature maps output from the max-pooling layer of the standard model. **(C)** Grad-Cam method for visualization of the tokens of the standard model. **(D)** Directly map the attention of the classification token on feature tokens to the original image.

Figure 12 provides the results of the visualization of the regions of interest to the model during the classification process. We chose ResNet50 to compare with the standard model and three visualization schemes. For the convolutional or pooling layer-based scheme, we chose the output of the last convolutional layer of layer2 of ResNet50 and the output of the first pooling layer of the standard model because they both output feature maps with a width of 28. In the tokens-based visualization scheme, we selected the output of the first LN layer in the last transformer encoder of the standard model. In the attention matrix-based visualization scheme, we combined the attention matrix of all transformer encoders in the entire model. By comparing Figures 12A,B, as can be seen, in field settings with complex backgrounds, ResNet50 has a large amount of attention scattered in the background. In contrast, the attention of the standard model is mainly focused on the leaf surface. Figure 12C shows that the attention is more refined when representing features based on tokens, effectively suppressing the background information and focusing more on the leaf surface lesions. Figure 12D shows the attention distribution of classification token to other feature tokens, which is consistent with the area of attention of the model, which also shows that the MSA calculation mechanism that does not increase the number of model parameters effectively enhances the attention of the model to crucial information and suppresses the useless background noise information.

## Discussion

The common CNNs represent the feature information of an image *via* feature maps, and deepening the depth of the network can generally achieve better performance, but this also increases the number of model parameters and computational effort. They are more suitable for object recognition. The pixels where these objects are located usually do not have similarities, and the overall pixels composition of the pattern presents the features of the target object. For maize leaf disease recognition, the pixels where the lesions are located usually have similarities (reflected in the RGB values), which requires a feature representation with higher resolution rather than the feature maps of CNNs. Since the feature maps increases with the number of channels but decreases in width as the network feeds forward. The relationship between lesions information and receptive field becomes blurred. There is no correlation computed between the lesions, so increasing the number of network layers will only bring a slight increase in recognition rate while also increasing the volume and complexity of the network. The model used in this article is entirely different from CNNs in that it is based on tokens to represent the visual information of local areas of the image. Stage 1 encodes the visual information of the receptive field into a matrix of feature tokens. The subsequent network does not perform any compression of this matrix. However, it continuously computes the correlation (attention) between tokens by MSA, making the network pay more attention to information about the lesions useful for classification and suppressing the noisy information in the background. We demonstrated this idea from this article’s theoretical, experimental, and visual analysis perspectives. Tokens represent the local feature information of images, and self-attention calculates the correlation of local information, which is more suitable for maize leaf disease identification in complex background. Therefore, guided by the above analysis, we designed a more reasonable model that achieves the best performance with minimal computational cost and number of parameters compared with other mainstream CNNs. However, our model has some limitations. The token (i.e., a single vector) dimension is a hyperparameter. As it increases, the feature information can be represented more abundantly, increasing the attention matrix’s scale. Large-scale matrix operations can rapidly increase the computational complexity of the model. Many researchers are now actively working to overcome this challenge (Carion et al., 2020; Liu et al., 2021; Touvron et al., 2021).

In addition, the results above indicate that convolution method outperforms the patch embedding method in encoding maize disease features into feature tokens. Convolution kernel as receptive field extracts visual information by sliding of itself. Two slides of the receptive field have an overlapped area, associating the semantic information of the area. However, the patch embedding method cuts a complete image into many irrelevant patches and directly encodes these patches into tokens, leading to the semantic information of adjacent areas to be lost. Humans tend to process critical vision information instead of all receptive field information, which is mainly limited by the brain’s inability to process massive information simultaneously. The mechanism by which humans process visual information is consistent with our model based on the attention mechanism, and they both prefer critical information.

In the field of plant disease identification, the hyperspectral imaging technology is usually used for object detection because the difference in reflectance of plant disease features is slight (Yue et al., 2015; Polder et al., 2019; Wang D. et al., 2019). The investigation of Nagasubramanian et al. (2019) demonstrated that soybeans infected the charcoal rot are more sensitive than healthy soybeans in the wavelengths of visible spectra (400–700 nm). Yang et al. (2021) have achieved good results in the Citrus Huanglongbing detection task by fusing hyperspectral data in CNNs using a multimodal approach. Recent research has shown that the transformer architecture is better suited for multimodal tasks (Frank et al., 2021; Zhang et al., 2021). We will conduct research by extending our model to combine with multimodal approaches for crop disease identification and detection in complex backgrounds in future.

## Data Availability Statement

The raw data supporting the conclusions of this article will be made available by the authors, without undue reservation.

## Author Contributions

XQ and KL conceived the study and wrote the manuscript. XQ implemented the algorithm. LC and CZ described the diseases and provided the dataset. All authors contributed to the article and approved the submitted version.

## Conflict of Interest

The authors declare that the research was conducted in the absence of any commercial or financial relationships that could be construed as a potential conflict of interest.

## Publisher’s Note

All claims expressed in this article are solely those of the authors and do not necessarily represent those of their affiliated organizations, or those of the publisher, the editors and the reviewers. Any product that may be evaluated in this article, or claim that may be made by its manufacturer, is not guaranteed or endorsed by the publisher.
